# Autoregulation of Cerebral Blood Flow During 3-h Continuous Cardiopulmonary Resuscitation at 27°C

**DOI:** 10.3389/fphys.2022.925292

**Published:** 2022-06-09

**Authors:** Sergei Valkov, Jan Harald Nilsen, Rizwan Mohyuddin, Torstein Schanche, Timofei Kondratiev, Gary C. Sieck, Torkjel Tveita

**Affiliations:** ^1^ Anaesthesia and Critical Care Research Group, Department of Clinical Medicine, UiT the Arctic University of Norway, Tromsø, Norway; ^2^ Division of Surgical Medicine and Intensive Care, University Hospital of North Norway, Tromsø, Norway; ^3^ Department of Research and Education, Norwegian Air Ambulance Foundation, Drøbak, Norway; ^4^ Department of Physiology and Biomedical Engineering, Mayo Clinic, Rochester, MI, United States

**Keywords:** cerebral oxygen delivery, cerebral oxygen consumption, cerebral blood flow (CBF), cerebral perfusion pressure (CPP), cerebral autoregulation

## Abstract

**Introduction:** Victims of accidental hypothermia in hypothermic cardiac arrest (HCA) may survive with favorable neurologic outcome if early and continuous prehospital cardiopulmonary resuscitation (CPR) is started and continued during evacuation and transport. The efficacy of cerebral autoregulation during hypothermic CPR is largely unknown and is aim of the present experiment.

**Methods:** Anesthetized pigs (*n* = 8) were surface cooled to HCA at 27°C before 3 h continuous CPR. Central hemodynamics, cerebral O_2_ delivery (DO_2_) and uptake (VO_2_), cerebral blood flow (CBF), and cerebral perfusion pressure (CPP) were determined before cooling, at 32°C and at 27°C, then at 15 min after the start of CPR, and hourly thereafter. To estimate cerebral autoregulation, the static autoregulatory index (sARI), and the CBF/VO_2_ ratio were determined.

**Results:** After the initial 15-min period of CPR at 27°C, cardiac output (CO) and mean arterial pressure (MAP) were reduced significantly when compared to corresponding values during spontaneous circulation at 27°C (−66.7% and −44.4%, respectively), and remained reduced during the subsequent 3-h period of CPR. During the first 2-h period of CPR at 27°C, blood flow in five different brain areas remained unchanged when compared to the level during spontaneous circulation at 27°C, but after 3 h of CPR blood flow in 2 of the 5 areas was significantly reduced. Cooling to 27°C reduced cerebral DO_2_ by 67.3% and VO_2_ by 84.4%. Cerebral VO_2_ was significantly reduced first after 3 h of CPR. Cerebral DO_2_ remained unaltered compared to corresponding levels measured during spontaneous circulation at 27°C. Cerebral autoregulation was preserved (sARI > 0.4), at least during the first 2 h of CPR. Interestingly, the CBF/VO_2_ ratio during spontaneous circulation at 27°C indicated the presence of an affluent cerebral DO_2_, whereas after CPR, the CBF/VO_2_ ratio returned to the level of spontaneous circulation at 38°C.

**Conclusion:** Despite a reduced CO, continuous CPR for 3 h at 27°C provided sufficient cerebral DO_2_ to maintain aerobic metabolism and to preserve cerebral autoregulation during the first 2-h period of CPR. This new information supports early start and continued CPR in accidental hypothermia patients during rescue and transportation for in hospital rewarming.

## 1 Introduction

Rewarming of a number of accidental hypothermia patients with hypothermic cardiac arrest (HCA) has been reported with favorable neurologic outcome if cardiopulmonary resuscitation (CPR) was started during evacuation and continued during transport to a hospital equipped for rewarming using extracorporeal life support (ECLS) (Walpoth et al., 1997; Gilbert et al., 2000; Mark et al., 2012; Wanscher et al., 2012; Boue et al., 2014; Hilmo, Naesheim, and Gilbert 2014). This is in essential contrast to CPR for cardiac arrest during normothermic conditions where poor neurologic outcome remains one of the leading causes for the high mortality rate in this patient group (Madl and Holzer 2004; Nolan et al., 2008; Stub et al., 2011; Allen et al., 2012).

Delivery of sufficient amounts of oxygenated blood to support cerebral O_2_ consumption (VO_2_), depends on the existence of a potent cerebral autoregulation. This homeostatic mechanism provides constant cerebral blood flow (CBF) over a wide range of cerebral perfusion pressure (CPP), i.e., the difference between actual mean arterial blood pressure (MAP) and intra-cerebral hydrostatic pressure (ICP) (Armstead 2016). In cases with malfunctioning cerebral autoregulation, CBF is governed by the actual CPP, which may lead to either cerebral hypo- or hyper-perfusion, both of which may result in cerebral edema, secondary cerebral ischemia, brain injury, and poor outcome (Ono et al., 2013). Therefore, during CPR, where MAP is significantly reduced below that during spontaneous circulation, a functional cerebral autoregulation is of paramount importance. During normothermic CPR, the time spent before the return of spontaneous circulation is critical for cerebral autoregulation to recover. In 13 out of 18 patients resuscitated from normothermic cardiac arrest with a median time to return of spontaneous circulation of 8 min, cerebral autoregulation was either absent or impaired (Sundgreen et al., 2001). Nishizawa and Kudoh (1996). found impaired cerebral autoregulation in all eight patients resuscitated from out of hospital cardiac arrest included in their study, and suggested that the level of deterioration of cerebral autoregulation was closely related to the level of cerebral ischemic injury following cardiac arrest. Nosrati et al., (2017) reported that a functioning cerebral autoregulation existed only during the first 200 s of normothermic CPR after cardiac arrest in a pig model.

Knowledge of cerebral autoregulation during hypothermic CPR is crucial to improve the quality of prehospital CPR during rescue and evacuation of accidental hypothermia patients in HCA (Monsieurs et al., 2015; Truhlář et al., 2015). By using the latest CPR algorithm (Truhlář et al., 2015), we demonstrated (Nilsen et al., 2020) that the level of cardiac output (CO) provided by CPR replacing spontaneous circulation during cardiac arrest is largely unaffected by core temperature down to 27°C. Further, our previous study documented that hypothermic CPR may provide limited but sufficient global oxygen delivery (DO_2_) to maintain global aerobic metabolism during a 3-h period at 27°C (Nilsen et al., 2020). Together with the well documented temperature-induced lowering of metabolic rate, and subsequent reduction in VO_2_ and CO, these findings may explain the increased survival of accidental hypothermia patients following continuous CPR.

We hypothesize that the reduced VO_2_ during hypothermia, and the existence of an unaltered CO during 3 h of CPR, may preserve autoregulation of CBF. To test this hypothesis, we used a porcine model of 3 h of CPR during hypothermic (27°C) cardiac arrest. The animals were equipped for repeated measurements of CBF and central and regional hemodynamics. After calculating cerebral VO_2_, cerebral autoregulation was estimated by use of the static auto- regulatory index (sARI) (Armstead 2016) and the CBF/VO_2_ ratio (Mezrow et al., 1992).

## 2 Materials and Methods

### 2.1 Ethical Approval

The Norwegian Food Safety Authority approved the study (ref. number: 14/56323). Eight castrated male pigs (20–29 kg), 3 months, from NOROC stock were used. The animals received humane care following the Norwegian Animal Welfare Act. The animals were placed in pens for 2–5 days after arriving at the laboratory animal unit. They were fed twice daily and had free access to water.

### 2.2 Anesthesia and Instrumentation

After an overnight fast, anesthesia was induced by an intramuscular bolus of ketamine hydrochloride 20 mg/kg (Ketalar, Pfizer Norge AS, Oslo, Norway), midazolam 30 mg (B. Brown Melsungen AG, Germany), and atropine 1.0 mg (Takeda AS, Asker, Norway). After transfer to the experimental laboratory, an ear vein catheter was inserted, and a bolus injection of fentanyl 10 μg/kg (Fentanyl-Hameln, Hameln Pharmaplus GMBH, Hameln, Germany) and pentobarbital-sodium 10 mg/kg (Ås production lab., Ås, Norway) was given. After tracheostomy, a continuous infusion of fentanyl 20 μg/kg/h, midazolam 0.3 μg/kg/h pentobarbital-sodium 4 mg/kg/h along with Ringer’s acetate 9 ml/kg/h in the right external jugular vein was started and maintained throughout the experiment. Neuromuscular blockers were not used at any time. Animals were ventilated without positive end-expiratory pressure (Siemens Servo 900D, Solna, Sweden). The fraction of inspired O_2_ was adjusted to maintain arterial PO_2_ > 10 kPa, and alveolar ventilation was adjusted to keep PaCO_2_ of 4.5–6.0 kPa uncorrected for temperature. Arterial blood gases were analyzed (ABL800 FLEX; Radiometer medical, Copenhagen, Danmark) to confirm adequate ventilation. After the experiment, animals were euthanized with an i.v. bolus of pentobarbital and 20 ml of potassium chloride.

A 6 F fluid-filled pigtail catheter (Cordis Corporation, Miami, FL, United States) was introduced into the right common carotid artery through a 10F Super Arrowflex (Arrow International Inc., Reading, PA, United States) introducer for microsphere injections. Pulmonary artery pressure (PAP), central venous pressure (CVP), core temperature measurements, and determination of mixed venous and venous blood gases were enabled by introducing a 7F pulmonary artery catheter (Edwards Lifesciences LLC, Irvine, CA, United States) to the pulmonary trunk *via* the right external jugular vein. A single dose of 5000 IU heparin was given after placement of a thermodilution catheter. The tip of another 7F Swan—Ganz thermodilution catheter was positioned in the aortic arch *via* the left femoral artery for arterial blood gas analysis, mean arterial pressure (MAP) recordings, and collection of a reference blood sample for the microsphere technique. A 18G central venous catheter (Arrow International Inc., Reading, PA, United States) was introduced cranially into the left external jugular vein and advanced to the jugular bulb for blood sampling. A 14F urinary bladder catheter was introduced *via* a lower abdominal incision for continuous monitoring of urinary output.

### 2.3 Experimental Protocol

Following instrumentation and 30 min stabilization period, baseline hemodynamic recordings were made. Subsequently, all animals were immersion-cooled in ice water to 27°C. At a blood-temperature of 27°C, HCA was induced by stimulating the epicardial surface with an alternating current (5–20 mA, 6 Hz, and 30 V) *via* a 15 cm long needle electrode inserted in the epigastric area and directed towards the apex of the heart and guided by suctioning of blood from the left ventricle. HCA was defined as the appearance of ventricular fibrillation (VF) on ECG simultaneous with the absence of fluctuations in arterial pressure. After CA for 90 s CPR was started, utilizing a chest compression device (LUCAS™ chest compression system, Physio-Control Inc., Lund, Sweden), and continued for 180 min. The piston on the device was equipped with a suction cup to provide active decompression with a continuous mode compression/decompression duty cycle of 50 ± 5% at a rate of 100 ± 5 compressions/min, with a compression depth of 4–5 cm.

Data sampling for the evaluation of hemodynamic variables, assessment of cerebral O_2_ transport and cerebral blood flow (CBF), by stable isotope-labeled microspheres (BioPhysics Assay Laboratory, BioPAL, Inc., Worcester, MA, United States), was performed at baseline, 32 and 27°C during cooling, and after 15, 60, 120, and 180 min of CPR. After termination of CPR, brain tissue biopsies were taken to calculate CBF at the above-mentioned time points.

### 2.4 Immersion Cooling

Animals were cooled by circulating cold water (5°C) in combination with ice slush to a level of two-thirds of the animal immersed in a waterproof reservoir mounted on the top of the operating table. The head was placed on a cushion and not immersed in cold water or covered with ice slush. Blood temperature was monitored via the thermistor on the pulmonary artery catheter (Edwards Lifesciences, Irvine, CA, United States). After reaching a blood core temperature of 28°C, the cold water was drained from the reservoir, and subsequently the core temperature dropped to 27°C. Careful warming was performed by positioning the operation lamps closer to the animal to prevent a further drop in core temperature.

### 2.5 Data Sampling

Each data sampling lasted about 10–15 min and was carried out in the following order: 1) mean arterial blood pressure (MAP), central venous pressure (CVP), and intracranial hydrostatic pressure (ICP). 2) Core temperature, diuresis, and respirator settings, 3) Blood sampling from the catheters placed in the femoral artery, pulmonary artery and both right and left jugular veins. 4) Injection of isotope-labeled microspheres into the left ventricle and simultaneous collection of a reference blood sample from aortic arch.

### 2.6 Stable Isotope Labeled Microsphere Technique for Counting Cerebral Blood Flow

Stable isotope-labeled microspheres were used to calculate regional blood flow in the brain (Christopher P. Reinhardt et al., 2000). The technique was described in detail in our previous study (Valkov et al., 2019). Tissue samples for determination of CBF were taken from the right and left temporal lobe, right and left cerebellum, and the hippocampal area.

Calculation of CBF (expressed in ml/min/g) by microsphere activity was conducted using the following equation: Q = (Tis_CPM_ × Q_ref_)/(Ref_CPM_ × g) where Q is blood flow in ml/min, Tis_CPM_ is the number of radioactive counts in the tissue sample in counts per min, Q_ref_ is the reference flow rate in ml/min, Ref_CPM_ is the number of radioactive counts in the reference blood sample in counts per min, and g is weight of the tissue sample.

### 2.7 Calculations

Cardiac output (CO) was calculated using the following formula: CO = Q_ref_ × Tot_CPM_/Ref_CPM_/1,000*,* where Q_ref_ is the reference flow rate in ml/min, Tot_CPM_ is total activity of injected microspheres, and Ref_CPM_ is an activity of the microspheres in the reference blood sample in counts per min. Mean cerebral blood flow was determined as average flow in the five different brain areas at the given time point. Cerebral vascular resistance (CVR) was calculated as (MAP − ICP)/CBF. Cerebral perfusion pressure (CPP) was calculated as MAP − ICP. The O_2_ content values were calculated according to the formula: SO_2_ × Hb × (1.34 × 10^−2^) and expressed as ml O_2_/100 ml blood. Cerebral DO_2_ was calculated as the product of mean CBF and arterial O_2_ content/g brain tissue. Cerebral VO_2_ was calculated as the product of mean CBF and the arterio-venous difference in O_2_ content/g brain tissue. The O_2_ extraction ratio (O_2_ER) was calculated as the ratio of VO_2_ to DO_2_.

### 2.8 Assessment of Cerebral Autoregulation

A static method to assess cerebral autoregulation was used (Armstead 2016). The steady state response in cerebral autoregulation to initiation of CA, and the subsequent CPP challenge, was assessed by analyzing changes in CBF after 15 min and each h during 3-h period of CPR compared to during spontaneous circulation at 27°C. The level of intact cerebral autoregulation was assessed by quantifying the static autoregulatory index (sARI) using the following formula: sARI = % ΔCVR/% ΔCPP = [(CVR_CPR_ − CVR_spontaneous circ. 27°C_)/CVR_spontaneous circ. 27°C_]/[(CPP_CPR_ − CPP _spontaneous circ. 27°C_)/CPP _spontaneous circ. 27°C_]. This index reflects the reactivity of the brain vessels to adjust to a reduced CPP. Thereby, if the proportional change in CPP matches proportional change in CVR, CBF does not change. A functioning cerebral autoregulation is documented to be present when sARI is between 0.4–1.0 (Lee et al., 2011).

In addition, the ratio CBF/cerebral VO_2_ was used to estimate the relationship between changes in CBF and cerebral O_2_ uptake (Mezrow et al., 1992). We assumed that the baseline value, created using data from spontaneous circulation at 38°C, represented normal cerebral autoregulation, i.e., an optimal relationship between cerebral blood flow and metabolism.

### 2.9 Statistical Analyses

Statistical analyses were performed using the SigmaPlot statistical software version 14 [Systat Software Inc., (SSI), Richmond, CA, United States]. Intragroup comparisons were performed by one-way repeated-measures analysis of variance (ANOVA) if a normal distribution of data was observed. Otherwise, the Friedman repeated measures ANOVA on ranks was used. If significant differences were found by ANOVA, Dunnett’s posthoc test was used to compare values within a group vs. 38°C or 27°C baseline. The level of significance was set at *p* ≤ 0.05. Data are means and SD.

## 3 Results

All animals had spontaneous circulation during cooling to 27°C before HCA was induced. Due to the chest compression device, multiple costal fractures were observed in all animals, and sternal fractures were observed in some animals after termination of the experiments. The cooling rate with immersion to 27°C was 6 ± 2.7°C/h. Occassional episodes of visible shivering were observed in every pig during cooling at different temperatures but were suppressed by an i.v. bolus of fentanyl.

### 3.1 Cooling to 27°C With Spontaneous Circulation

#### 3.1.1 Hemodynamics

**FIGURE 1:**
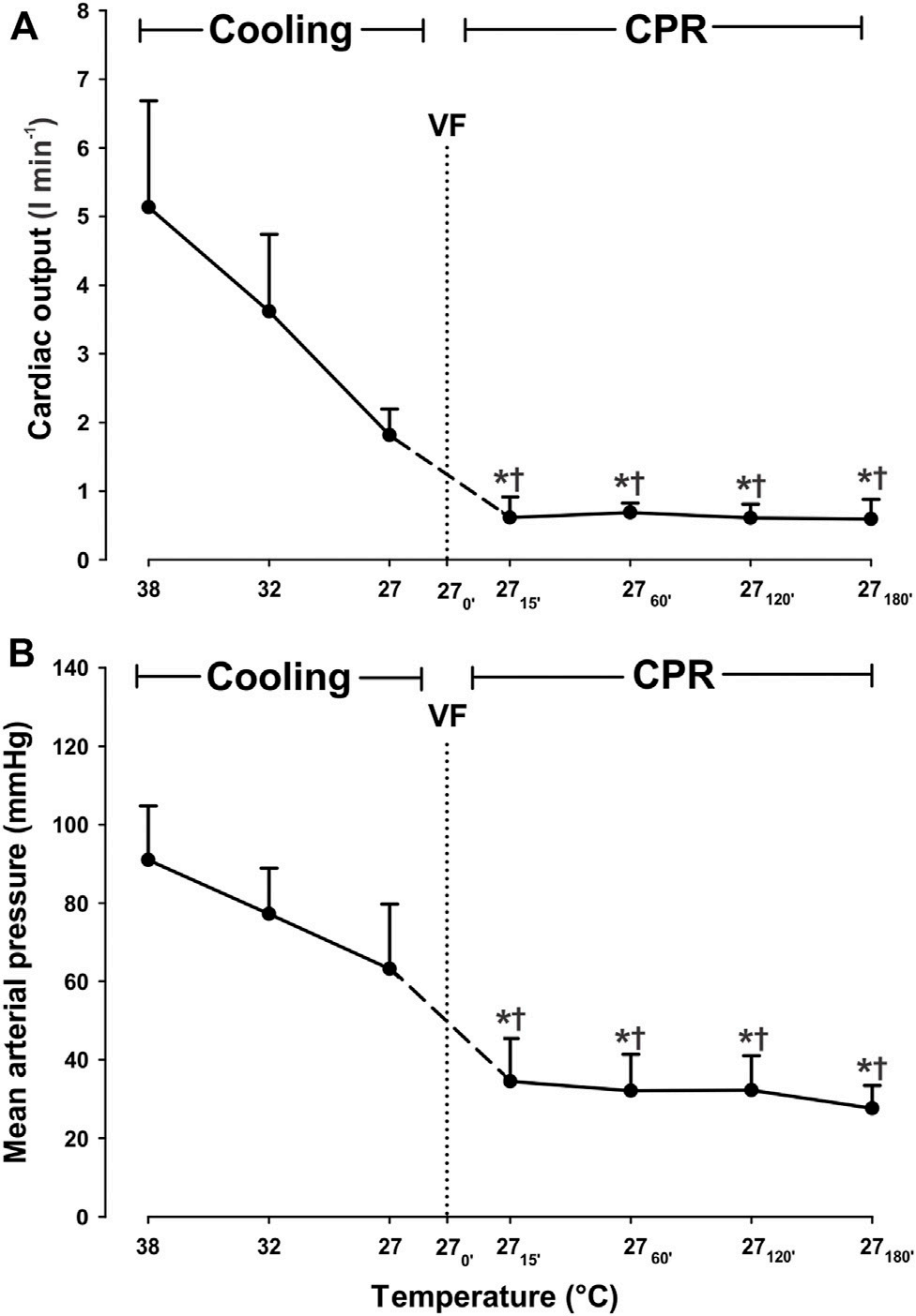
Hemodynamic variables during cooling with sinus rhythm and 3 h CPR. **(A)** cardiac output, **(B)** mean arterial pressure. Values are mean ± SD (*n* = 8). *p < 0.05 vs. intragroup 38°C baseline; ^†^ *p* < 0.05 vs. intragroup spontaneous circulation at 27°C VF - ventricular fibrillation.

A linear reduction in CO and MAP (Figure 1) was seen during cooling to 27°C, but these changes did not reach statistical significance. CVP and ICP remained stable during cooling from 38 to 27°C (Table 1).

**TABLE 1:** Values are mean ± SD (*n* = 8).

	BL_38°C_	32°C	27°C	CPR_15 min_	CPR_1h_	CPR_2h_	CPR_3h_
CVP (mmHg)	6 ± 2	6 ± 1	6 ± 2	13 ± 2*†	14 ± 3*†	14 ± 3*†	12 ± 3*†
ICP (mmHg)	13 ± 3	14 ± 4	14 ± 4	18 ± 2	20 ± 3	17 ± 3	16 ± 4
MAP (mmHg)	91 ± 14	77 ± 12	63 ± 17	35 ± 11*†	32 ± 9*†	32 ± 9*†	28 ± 6*†
P_a_CO_2_ (kPa)	4.5 ± 0.5	5.3 ± 0.7	6.0 ± 0.8*	5.5 ± 1.2	6.2 ± 1.6*	6.7 ± 1.2*	7.4 ± 1.8*†
pH	7.54 ± 0.05	7.48 ± 0.04	7.42 ± 0.03*	7.41 ± 0.05*	7.34 ± 0.08*†	7.27 ± 0.06*†	7.2 ± 0.09*†
Lactate (mmol/L)	1.0 ± 0.7	0.7 ± 0.1	0.6 ± 0.1	1.1 ± 0.5	1.9 ± 0.8	3.9 ± 1.7*†	5.7 ± 2.4*†
sARI				0.9 ± 0.2	0.7 ± 0.4	0.6 ± 0.8	0.1 ± 1.8

Abbreviations: CVP, central venous pressure; ICP, intracranial pressure; MAP, mean arterial pressure; P_a_CO_2_, partial arterial pressure of carbon dioxide; sARI, static autoregulatory index.

*p < 0.05 vs. intragroup 38°C baseline; ^†^ *p* < 0.05 vs. intragroup spontaneous circulation at 27°C.

#### 3.1.2 Cerebral O_2_ Transport

**FIGURE 2:**
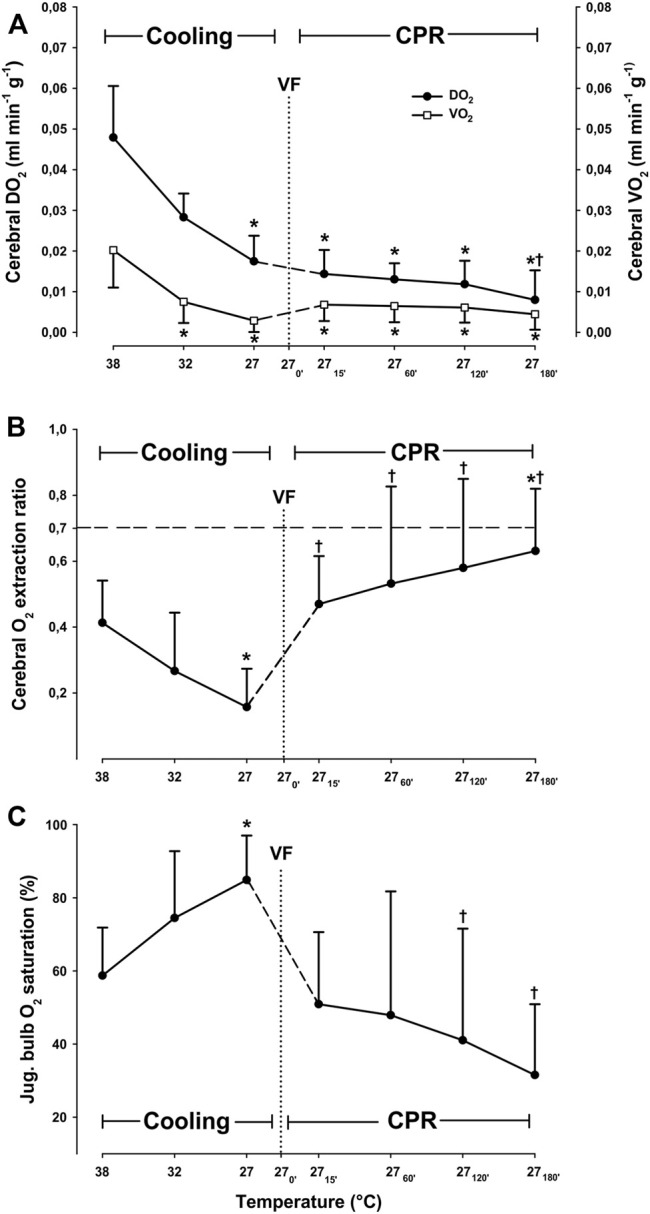
Cerebral oxygen transport, **(A)** cerebral O_2_ extraction ratio **(B)**, and jugular bulb O_2_ saturation **(C)** during cooling and 3 h CPR at 27°C (*n* = 8). Values are mean ± SD (*n* = 8). *p < 0.05 vs. intragroup 38°C baseline; ^†^ *p* < 0.05 vs. intragroup spontaneous circulation at 27°C.

Cooling reduced cerebral O_2_ delivery (DO_2_) and consumption (VO_2_) linearly (Figure 2), and at 27°C both DO_2_ and VO_2_ were significantly reduced from 0.049 ± 0.013 to 0.016 ± 0.004 ml^∗^min^−1^^∗^g^−1^ (−67.3%) and from 0.022 ± 0.009 to 0.003 ± 0.003 ml^∗^min^−1^^∗^g^−1^ (−86.4%), respectively. The O_2_ extraction ratio (ER; VO_2_/DO_2_) was significantly reduced (−55%) at 27°C when compared to its pre-hypothermic baseline values, whereas cerebral venous O_2_ saturation (S_v_O_2_) increased from 58.7% ± 13.1 at 38°C to 84.9% ± 12.1 at 27°C (+44.6%).

#### 3.1.3 Cerebral Blood Flow

**FIGURE 3:**
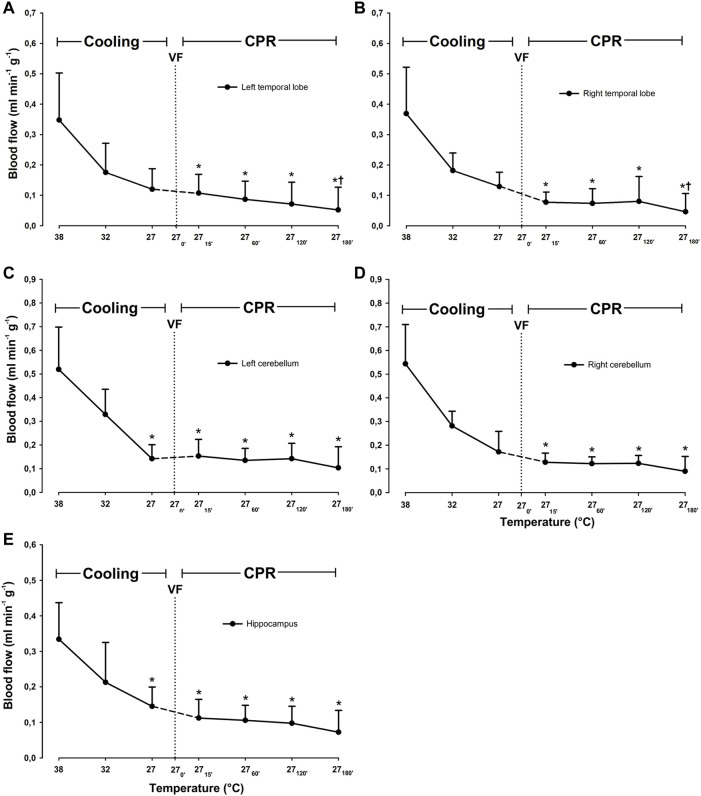
Cerebral blood flow during cooling and 3 h CPR at 27°C. **(A)** left temporal lobe blood flow, **(B)** right temporal lobe blood flow, **(C)** left cerebellum blood flow, **(D)** right cerebellum blood flow, **(E)** hippocampus blood flow. Values are mean ± SD (*n* = 8). *p < 0.05 vs. intragroup 38°C baseline; ^†^ *p* < 0.05 vs. intragroup spontaneous circulation at 27°C.

**FIGURE 4:**
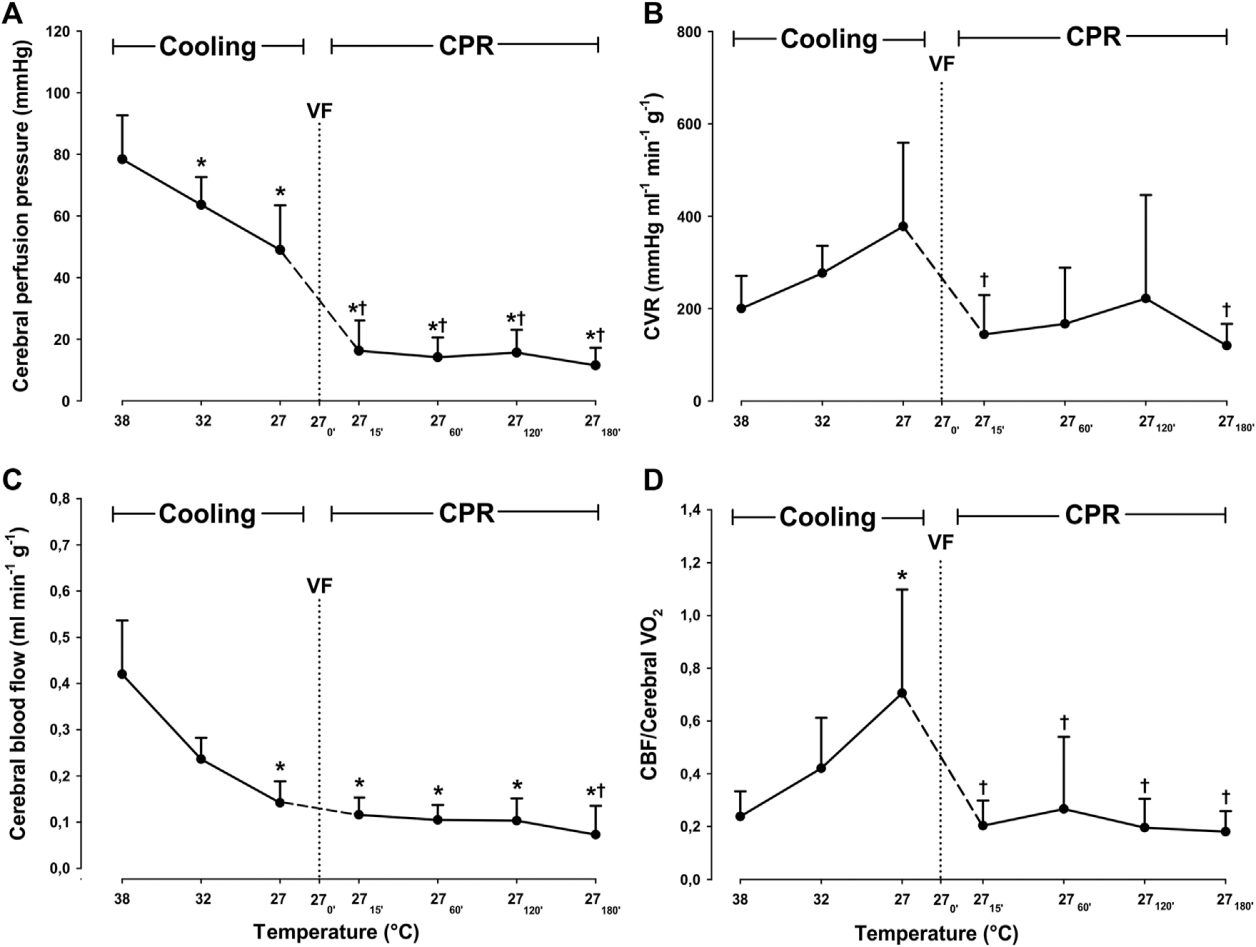
Determinants of cerebral autoregulation, during cooling and 3 h CPR at 27°C. **(A)** cerebral perfusion pressure, **(B)** cerebral vascular resistance (CVR), **(C)** mean cerebral blood flow (CBF), **(D)** CBF/cerebral O_2_ consumption ratio. Values are mean ± SD (*n* = 8). *p < 0.05 vs. intragroup 38°C baseline; ^†^ *p* < 0.05 vs. intragroup spontaneous circulation at 27°C.

Compared to 38°C, cooling to 27°C caused a significant reduction in blood flow only in the left cerebellum, from 0.52 ± 0.178 to 0.142 ± 0.059 ml^∗^min^−1^*g^−1^ (−61.5%), and hippocampus from 0.334 ± 0.103 to 0.145 ± 0.054 ml*min^−1^*g^−1^ (−56.6%) (Figure 3). Compared to 38°C, mean CBF was significantly reduced during spontaneous circulation at 27°C, from 0.42 ± 0.12 to 0.14 ± 0.05 ml*min^−1^*g^−1^ (−66.2%) (Figure 4).

### 3.2 Continuous 3-h Period of CPR at 27°C

#### 3.2.1 Hemodynamics

After 15 min of CPR, both CO and MAP were significantly decreased compared to corresponding values during both spontaneous circulation at 38 and at 27°C (Figure 1). When compared to corresponding levels during spontaneous circulation at 38°C, CO was reduced from 5.1 ± 1.5 to 0.6 ± 0.3 L/min (−88.2%), and MAP was reduced from 91 ± 14 to 35 ± 11 mmHg (−61.5%). However, both CO and MAP remained statistically unchanged at these reduced levels during the remaining 3-h period of CPR. After 15 min CPR at 27°C, a modest but still significant increase in CVP (Table 1), from 6 ± 2 to 13 ± 2 mmHg, took place when compared to during spontaneous circulation both at 38°C and at 27°C. CVP remained unchanged at this increased level throughout the 3-h period of CPR at 27°C.

#### 3.2.2 Cerebral Blood Flow

During the first 2 h of CPR, cerebral blood flow remained significantly unchanged in all areas of the brain evaluated when compared to 27°C spontaneous circulation (Figure 3). After 3 h of CPR, blood flow was significantly reduced in both right and left temporal lobes (0.13 ± 0.05 to 0.05 ± 0.06, and 0.12 ± 0.07 to 0.05 ± 0.07 ml*min^−1^*g^−1^, respectively) as compared to corresponding values during spontaneous circulation at 27°C. Compared to mean CBF during spontaneous circulation at 27°C, mean CBF during the first 2 h of CPR remained at this reduced level (Figure 4). By 3 h of CPR, a significant reduction in mean CBF from 0.14 ± 0.05 ml*min^−1^*1 g^−1^ to 0.07 ± 0.06 ml*min^−1^*g^−1^ (−50%) was observed.

#### 3.2.3 Cerebral O_2_ Transport

After 15 min of CPR at 27°C, cerebral DO_2_ was reduced by 17.6%, when compared to spontaneous circulation at 27°C (Figure 2). However, this reduction in cerebral DO_2_ was not statistically significant, and cerebral DO_2_ remained at the same reduced level for the next 2 h of CPR. After 3 h of CPR, a significant reduction in cerebral DO_2_ from initial 0.017 ± 0.006 at 27°C, to 0.008 ± 0.008 ml*min^−1^*g^−1^ (−53%) had taken place. After 15 min of CPR at 27°C, cerebral VO_2_ increased from 0.003 ± 0.003 to 0.007 ± 0.004 (+43%). Cerebral VO_2_ remained statistically unchanged throughout 3-h period of CPR compared to the corresponding level during spontaneous circulation at 27°C. Cerebral O_2_ extraction ratio (ER; VO_2_/DO_2_) returned to its pre-hypothermic level after 15 min of CPR and remained unchanged during the first 2 h of CPR but increased significantly from 0.41 ± 0.13 to 0.63 ± 0.19 (+35%) after 3 h of CPR. However, the cerebral ER was still below the reported critical extraction ratio (ER_crit_) of 0.7 (Leach and Treacher, 2002) during 3 h of CPR. Jugular bulb venous blood O_2_ saturation returned to its pre-hypothermic baseline level and remained unchanged during the 3-h period of CPR, indicating an increase in O_2_ extraction.

#### 3.2.4 Cerebral Autoregulation

After 15 min of CPR, CPP was significantly reduced when compared to both spontaneous circulation at 38 and at 27°C, but during the rest of the 3-h period of CPR, CPP remained statistically unchanged at this reduced level (Figure 4). When compared to spontaneous circulation at 38°C, cerebral vascular resistance (CVR) remained statistically unchanged during cooling and the subsequent 3 h of CPR. However, if compared to the level during spontaneous circulation at 27°C, CVR was significantly reduced after 15 min of CPR from 378 ± 181 to 144 ± 85 mmHg*mL^−1^*g^−1^*min^−1^ (−60.1%), and after 3 h of CPR to 109 ± 43 mmHg*mL^−1^*1 g^−1^*min^−1^ (−71.2%).

As a consequence of these changes in CVR and CPP, the static autoregulatory index (sARI) changed during the 3-h period of CPR at 27°C (Table 1): During the first 2 h, sARI remained above its lower limit (0.4); after 15 min sARI was 0.9 ± 0.2, after 1 h 0.7 ± 0.4 and after 2 h 0.6 ± 0.8, indicating the presence of a functioning autoregulation (Strebel et al., 1995; Lee et al., 2011). After 3 h of CPR, sARI fell to 0.1 ± 1.8, indicating impaired autoregulation.

During cooling, the CBF/Cerebral VO_2_ ratio was significantly increased when compared to baseline values at 38°C. However, after 15 min of CPR and during the subsequent 3-h period of CPR, the CBF/Cerebral VO_2_ ratio returned to the 38°C baseline level.

## 4 Discussion

This study demonstrated that a continuous 3-h period of CPR for hypothermic cardiac arrest at 27°C maintains cerebral blood flow, cerebral perfusion pressure, and O_2_ metabolism. These findings gain support when calculating the static autoregulatory index, ARI, which clearly indicates that cerebral autoregulation is functioning, at least during the first 2 h of the 3 h resuscitation period.

The protective effects of hypothermia on end-organ survival are well-recognized based on clinical emergency medicine reports (Gilbert et al., 2000; Mark et al., 2012; Wanscher et al., 2012). However, with respect to neurologic outcome after accidental hypothermia, existing reports of the effects of hypothermia on cerebral autoregulation are conflicting. The majority of preclinical CPB studies (Mezrow et al., 1992; Lee et al., 2011; Wang et al., 2019), as well as human studies from cardiac surgery (Preisman et al., 2005; Ono et al., 2013), suggest protective effects of mild hypothermia (36–34°C) on cerebral autoregulation in post-cardiac arrest states. In contrast, when mimicking the clinical scenario of accidental, deep hypothermia (<30°C), Gaasch et al. (2020) reported that although mild hypothermia did not affect indexes of autoregulation, temperatures below 34°C were associated with altered cerebral autoregulation.

There are two acknowledged methods to evaluate the efficacy of cerebral autoregulation *in vivo*; 1) static, and 2) dynamic methods (Armstead 2016). To monitor dynamic autoregulation, MAP or CPP have to be changed repeatedly during experiments by either thigh-cuff inflation/deflation (Aaslid et al., 1989) or by other methods (Diehl et al., 1995; Reinhard et al., 2006). The efficacy of autoregulation is then determined as the rate of decrease and return of CBF during interventions. In the present study, the steady-state relationship between changes in CPP and cerebral vascular resistance (CVR) was assessed, reflecting the reactivity of the brain vessels to a decrease in CPP, as the consequence of the HCA and subsequent CPR. This approach suits the application of the static method enabling calculation of the static cerebral autoregulation index (sARI), which indicates that a functioning cerebral autoregulation is present when sARI is between 0.4 and 1.0 (Lee et al., 2011). In our study, the sARI during the first 2 h of CPR was above 0.6, indicating the presence of a functioning, normal cerebral autoregulation during this interval.

In cases with altered autoregulation, CBF is determined by changes in MAP or CPP, which may lead to either hyper or hypo-perfusion of the brain (Ono et al., 2013). In the current study, there was a substantial decrease in CPP and MAP after the initiation of CPR, but during the subsequent 2-h period, there was no significant reduction in mean CBF. This indicates that cerebral autoregulation was preserved despite a significant reduction in both CO and MAP (Figure 4). In support of a functioning cerebral autoregulation during the first 2 h of CPR at 27°C, we measured a uniform distribution of CBF in five specific areas of the brain, in addition to the existence of a stable ICP. A stable ICP (within physiologic levels) also indicates the absence of cerebral hyperemia (Kelly et al., 1996), which otherwise may take place as a consequence of ischemic damage of the capillary integrity or damage to the blood-brain barrier.

During normothermic CPR a low CO does not meet tissue O_2_ demands, which quickly results in tissue hypoxia and acidosis, causing a reduction in vascular tone that may lead to vasoparalysis (Takagi et al., 1977). Physiologically, this is expected to cause volume overload in central capacitance vessels, leading to an increase in central venous pressure (CVP). The latter may be followed by an increase in ICP and, thus, to a decrease of CPP. Ameloot et al. (2016) found that after the return of spontaneous circulation with restitution of CO and MAP in post cardiac arrest patients, an elevated CVP resulted in a reduced DO_2_ which also correlated with a negative neurological outcome. In our previous study (Nilsen et al., 2020), a normothermic control group was included, which underwent CPR for 45 min. In this group, CVP increased from 7 to 43 mmHg by 15 min of CPR. In contrast, during CPR at 27°C, CVP increased from six to only 14 mmHg after 3 h, suggesting the existence of an antegrade blood flow and the absence of both venous congestion and cerebral hypoperfusion.

During spontaneous circulation, it is well documented that hypothermia slows metabolic rate, global DO_2_, and VO_2_ (Black, van Devanter, and Cohn 1976; Kondratiev et al., 2006; Valkov et al., 2019), which we also prevuiously reported during spontaneous circulation at 27°C (Valkov et al., 2019; Nilsen et al., 2020). However, when compared to spontaneous circulation at 27°C, the reduction in CO, MAP, and cerebral DO_2_ that occurred with CPR remained unchanged during the first 2 h of CPR. This supports our interpretation of a functioning cerebral autoregulation during this period. The present data also demonstrates that the significantly lower cerebral DO_2_ after 3 h of hypothermic CPR was compensated for by increased O_2_ extraction. The additional finding of a reduced S_v_O_2_ and a maintained cerebral VO_2_ also support the presence of a use- dependent O_2_ consumption also during the last h of CPR at 27°C. In order to further demonstrate the relationship between DO_2_ and VO_2_ during compromised circulation, we have utilized the O_2_ extraction ratio concept (ER = VO_2_/DO_2_) (Schumacker and Cain 1987). In general, in cases with a limited O_2_ delivery, ER approaches a critical value (0.7) at which VO_2_ becomes dependent on DO_2_, a situation which may cause imminent tissue hypoxia due to delivery-dependent O_2_ transport. It is known that this critical level does not change in hypothermia (Leach and Treacher 2002). However, in contrast to our previous finding of an elevated global ER (Nilsen et al., 2020), data from the current study show that cerebral ER did not exceed the critical level throughout the 3-h period of CPR at 27°C, indicating the presence of preserved use-dependent cerebral O_2_ transport.

Together with the lowering of sARI to 0.1 after 3 h of CPR, we also found that CBF was significantly reduced when compared to during spontaneous circulation at 27°C. It is, therefore, likely that cerebral autoregulation was impaired at this point. But, despite a malfunctioning cerebral autoregulation after 3 h of CPR, the reduced CBF appeared sufficient to provide a balance between O_2_ delivery and consumption. We believe that this was feasible due to the existence of an excess DO_2_ generated during spontaneous circulation at 27°C, before the initiation of CPR. To show the abundancy of cerebral DO_2_, we used the CBF/Cerebral VO_2_ ratio (Mezrow et al., 1992; Mezrow et al. 1994; Mezrow et al. 1995). The normothermic (38°C) value of the CBF/Cerebral VO_2_ ratio represents normal cerebral autoregulation, i.e., an optimal relationship between CBF and cerebral VO_2_. During spontaneous circulation at 27°C, CBF and cerebral DO_2_ exceeded cerebral VO_2_, indicating the presence of a “luxurious cerebral perfusion.” This interpretation is supported by the finding of a simultaneous increase in cerebral S_v_O_2_. After the start of CPR, the CBF/Cerebral VO_2_ ratio returned to pre-hypothermic baseline values. It remained unchanged for the next 3 h of CPR, indicating a functioning cerebral autoregulation during at least the first 2 h of CPR. However, the stable CBF/Cerebral VO_2_ ratio found after 3 h of CPR appears to be due to a functioning excess unloading of O_2_ from hemoglobin at 27°C (Figure 4).

### 4.1 Limitations

After 2 h of CPR at 27°C, calculations of sARI gave negative values in 2 out of 8 animals, indicating a malfunctioning autoregulation in these two animals. Due to these negative values causing an increase in variability in our dataset, the changes in sARI during continuous CPR remained statistically unchanged when compared to spontaneous circulation at 27°C.

## 5 Conclusion

The results of this study indicate that cerebral autoregulation was maintained, at least during the first 2 h of the continuous 3-h period of CPR at 27°C. This may represent an important background mechanism for the favorable neurologic outcome reported in accidental hypothermia patients treated with continuous CPR during transportation for in-hospital rewarming.

## Data Availability

The raw data supporting the conclusion of this article will be made available by the authors, without undue reservation.
